# Somatic mutation in human cerebellum illustrates neuron type-specific patterns of age-related mutation

**DOI:** 10.64898/2026.02.27.708647

**Published:** 2026-03-02

**Authors:** Kow Essuman, Yingxi Yang, Eitan Goodman, Christie N. Cambridge, Chunhui Cai, Zheming An, Shulin Mao, Monica Devi Manam, Benjamin Finander, Sattar Khoshkhoo, Liang Sun, August Yue Huang, Christopher A. Walsh

**Affiliations:** 1 Division of Genetics and Genomics, Manton Center for Orphan Disease Research, Boston Children’s Hospital, Boston, MA, USA.; 2 Department of Neurosurgery, Massachusetts General Hospital, Boston, MA, USA; 3 Department of Neurology, Mass General Brigham, Boston, MA, USA; 4 Departments of Pediatrics and Neurology, Harvard Medical School, Boston, MA, USA; 5 Broad Institute of MIT and Harvard, Cambridge, MA, USA; 6 Research Informatics, Department of Information Technology, Boston Children’s Hospital, Boston, MA, USA; 7 Biological and Biomedical Sciences Program, Harvard University, Cambridge, MA, USA.; 8 Howard Hughes Medical Institute, Boston Children’s Hospital, Boston, MA, USA

## Abstract

Human neurodegenerative disorders are characterized by exquisite specificity for neuronal types, but the basis of this is unknown. Here, we show that cerebellar granule neurons (GN)—the most abundant neuronal type in the human brain—accumulate somatic mutations in patterns highly distinct from cerebral cortical neurons, and more closely resembling oligodendroglia and other dividing cells. We find shared mutational signatures between normal aging GNs and medulloblastoma subtypes, suggesting the GN lineage as a tumor cell of origin. Whole-genome sequence of multiple single GNs from the same donor allowed analysis of specific times of neurogenesis, revealing a rich lineage tree that includes GNs that become postmitotic 2 years or more after birth, yet migrating postnatally to populate both the cerebellar vermis and the distant cerebellar hemisphere. Our results show that neuronal type-specific somatic mutation patterns enlighten normal development, cancer origins and potentially the cell type-specificity of neurodegeneration.

The human brain is estimated to contain as many as one thousand distinct neuronal types, defined by their morphology, connections, physiological activity, transcriptomic diversity (1, 2), and prominently the fact that they are postmitotic and do not undergo further cell division once formed (3). Despite their postmitotic state, cerebral cortical neurons show somatic mutations that reflect their cell lineage during development (4, 5), but also continue to accumulate mutations throughout their postmitotic life (6–8), at rates comparable to those of dividing cells such as hematopoietic stem cells (6, 9, 10). In cerebral cortical pyramidal neurons, where they have been studied, these age-related mutations appear to be related to DNA damage occurring in relation to gene transcription, since they are enriched in genes and regulatory sequences (5, 7, 8, 11), in contrast to somatic mutations in oligodendrocytes (OLs), which show little relation to transcription and a stronger relationship to cell cycle-related DNA replication (11).

Despite the potential importance of age-related somatic mutations in neurons for neurodegenerative disease (12–14), they have so far only been intensively studied in a single neuronal type, the excitatory pyramidal neurons of the cerebral cortex (12, 13). Therefore, it is unknown how the rules defined for cortical neurons may or may not apply to other neuronal types in the central nervous system. Degenerative diseases affecting the nervous system are as rule quite specific for cell types (15) emphasizing the need to study somatic mutation in other neuronal types.

Granule neurons (GNs) of the cerebellum are the most numerous neurons of the human brain, estimated to be more abundant than all other neurons in the brain combined, and have several distinctive features (16, 17). They have small nuclei and perikarya, and are formed relatively late in development, with animal studies suggesting that they continue to be formed for some time after birth (16–18). They derive from precursors of the rhombic lip, which migrate out tangentially over the outer surface of the incipient cerebellum, forming a cell-dense layer referred to as the external granule cell layer (EGL) (16, 17, 19). Classical histological studies in humans show that this EGL persists at least a year after birth (20–23), though the extent of formation of neurons after birth in human cerebellum is not known.

Most degenerative disorders that affect the pyramidal neurons of the cerebral cortex do not typically affect GNs (15). However, there are GN-specific forms of degeneration, and disorders of cerebellar development and cancer (24–26) that prompt a need for better understanding of the somatic mutation landscape in the cerebellum. Our analysis of age-related somatic mutation rates and patterns in GNs show widespread differences from cortical neurons and illustrate the unique development of the human cerebellum.

## Cerebellar GNs accumulate somatic mutations at distinct rates from cortical neurons

**Fig. 1.**
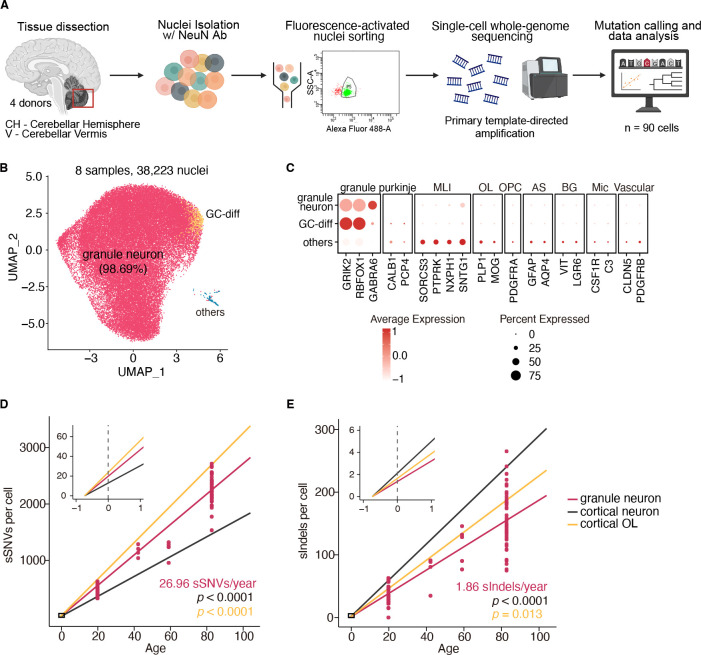
Genome-wide rates of somatic mutation accumulation in cerebellar GNs. A. Schematic of experimental design. GNs were isolated from postmortem human cerebellar tissue, followed by primary template amplification (PTA), whole-genome sequencing, and downstream variant analysis. B. Uniform manifold approximation and projection (UMAP) representation of snRNA-seq profiles generated from NeuN-sorted cerebellar samples. Major cell types and their relative proportions are labeled. GC-diff, granule cell-differentiating cells. C. Dotplot showing the mean expression of marker genes and the percentage of cells expressing them for each annotated cell type. MLI, molecular layer interneurons; OL, oligodendrocyte; OPC, oligodendrocyte precursor cell; AS, astrocyte; BG, Bergmann glia; Mic, microglia. D. sSNV burden as a function of age in GNs (red), cortical neurons (black), and cortical OLs (orange). Data points represent single GNs; trend lines reflect linear mixed-effects model; the inset shows an enlarged view of modeled early-life accumulation (fertilization to one year). P values compare GNs with cortical neurons and OLs (GN vs cortical neuron: *p* < 0.0001; GN vs cortical OL: *p* < 0.0001). E. sIndel burden analyzed as in (D). GN vs cortical neuron: *p* < 0.0001; GN vs cortical OL: *p* = 0.013.

We first purified NeuN+ nuclei from post-mortem human cerebellar hemisphere or vermis using fluorescent activated nuclei sorting (FANS) (Fig. 1A and fig. S1A). NeuN+ was chosen to enrich for neuronal populations, but to ensure our sorting strategy yielded mature GNs rather than their precursors, we performed single-nuclei RNA sequencing (snRNA-seq) on the sorted population (Fig. 1, A to C, and fig. S1B). We generated 38,223 nuclei from eight samples across four neurotypical individuals aged 19.8, 42.2, 59, 82.7 years (Fig. 1B, fig. S1B, table S1). We found that high quality cells from snRNA-seq yielded 99% GN purity with marker genes including *GRIK2* and *GABRA6* (27, 28) to confirm mature GN purity (Fig. 1, B and C). Less than 1% of sorted neurons lacked expression of *GABRA6* and these cells were classified as differentiating, immature GNs (Fig. 1, B and C, and fig. S1B).

To determine how somatic mutations accumulate with age in GNs, we performed single-cell whole genome amplification with primary template amplification (PTA) as previously described (8, 12), and performed whole genome sequencing (scWGS) on 90 single GNs from the four individuals (age 19.8, 42.2, 59, 82.7), targeting 30X coverage for each cell (range 19–63X coverage) (Fig. 1A). We then analyzed single genomes using our well established SCAN2 genotyper (8, 11) to accurately identify somatic single nucleotide variants (sSNVs) and somatic insertions-deletions (sIndels) from each cell (table S2 and S3). Somatic mutations in GNs were then compared with those in 56 cerebral cortical pyramidal neurons and 66 OLs previously sequenced from human prefrontal cortex (8, 11) (table S1).

Similar to cerebral cortical neurons and OLs, cerebellar GNs accumulate somatic sSNVs and sIndels with aging. The rate of sSNV accumulation in GNs is 26.96 sSNV/yr (95% confidence interval [CI]: 25.1–29.0), which is faster than that of cortical neurons 17.59 sSNVs/yr (CI: 16.1–19.1; for the difference, p = 3.9 × 10^−10^, Tukey-corrected t-test), but slower than cortical OLs 32.32 sSNVs/yr (CI: 30.6–34.0; p = 1.3 × 10^−7^, Tukey-corrected t-test) (Fig. 1D). In contrast, sIndels accumulate at a slower rate in GNs (1.86 sIndels/yr, CI: 1.6–2.1) compared to both cortical neurons (2.88 sIndels/yr, CI: 2.7–3.1; p = 4.5 × 10^−9^, Tukey-corrected t-test), and cortical OLs (2.24 sIndels/yr, CI: 2.0–2.5; p = 0.013, Tukey-corrected t-test) (Fig. 1E). These trends persisted after controlling for multiple quality metrics (fig. S2). Analysis of mutation burden and mutational spectra across cerebellar regions revealed no marked differences between GNs isolated from the midline vermis region, or the more laterally situated, functionally distinct, cerebellar hemispheres (fig. S3). Altogether, these findings suggest that different cell types in the human brain, even among neurons, accumulate somatic mutations at different rates, and hence may have unique vulnerabilities or response mechanisms to genomic damage.

## sSNV signatures of cerebellar GNs differ from that of cortical neurons

**Fig. 2.**
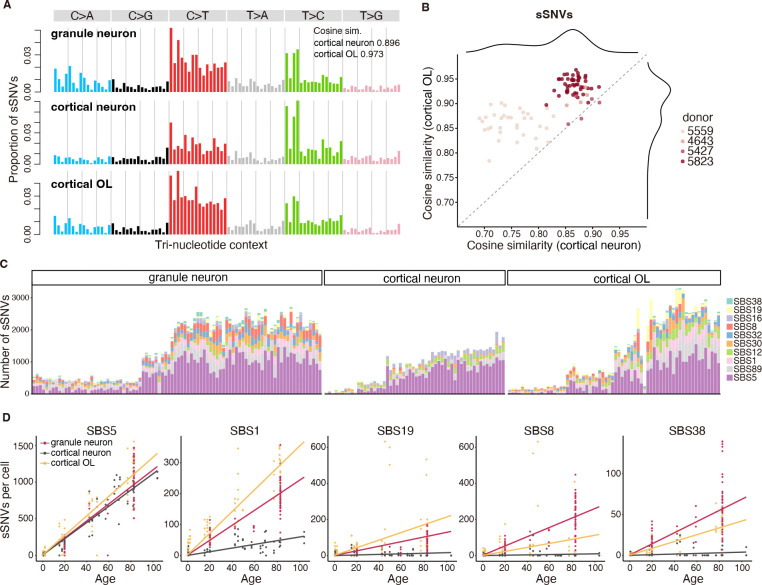
Cerebellar GNs show stronger age-associated increases in cell-division-related mutational signatures than cortical neurons. A. sSNV mutational spectrum of the three cell types, with cosine similarities shown for GNs relative to cortical neurons and OLs. B. Cosine similarity of sSNV spectrum from each GN relative to cortical neurons and OLs. Points represent single GNs colored by subject. C. Decomposition of sSNV burden into COSMIC SBS signatures in GNs, cortical neurons, and OLs. Subjects are ordered by increasing age from left to right. D. Contributions of the selected COSMIC SBS signatures to the sSNV burden in GNs, cortical neurons, and OLs.

To better understand the potential mutational processes occurring with age and to provide clues as to their potential underlying mechanisms, we performed mutational spectrum analysis of both sSNVs and sIndels of GNs. GNs showed a significant increase in C>A (p = 5.3 × 10^−21^, two-tailed Wilcoxon test) and C>T (p = 1.9 × 10^−10^, two-tailed Wilcoxon test) mutations compared to cortical neurons (Fig. 2A). Notably, comparison of mutational spectra indicated that the mutational spectrum of GNs was more similar to OLs (cosine similarity 0.973) than to cortical neurons (cosine similarity 0.896) (Fig. 2, A and B).

To disentangle the underlying molecular processes responsible for the accumulation of mutations in GNs, we performed COSMIC signature decomposition analysis as previously described (29), combining mutational patterns refitting with stepwise regression to prioritize the best-fitting signatures (30). This revealed an unexpected accumulation of the cell-division-associated mutational signature SBS1 in aging GNs (Fig. 2, C and D). SBS1 is enriched in rapidly dividing cells including cancer cells and is thought to reflect enzymatic or spontaneous deamination of 5-methylcytosine to thymine during the cell cycle, and is enriched at CpG islands in the genome (6, 31, 32). We found GNs from the aged individual showed markedly elevated SBS1 exposure relative to younger individuals, despite the postmitotic nature of GNs. On the other hand, age-matched cortical neurons do not show such high rates of SBS1 accumulation (Fig. 2, C and D; for the difference between GNs and cortical neurons, p = 2.7 × 10^−14^, Tukey-corrected t-test). The rate of SBS1 accumulation in GNs however appears to be slower than that in OLs (Fig. 2, C and D; p = 9.7 × 10^−13^, Tukey-corrected t-test). Other mutational signature patterns commonly seen in dividing cells also increased with age in GNs, including SBS19 (for the difference between GNs and cortical neurons, p = 2.5 × 10^−3^; GNs and OLs, p = 0.01; Fig. 2D) and SBS32 (GNs and cortical neurons, p = 4.4 × 10^−7^; GNs and OLs, p = 0.27, Tukey-corrected t-test; fig. S4B), observed in blood, liver and brain cancers (33–36). Our data therefore reveals shared mutational signatures and patterns between GNs and OLs, which suggests shared underlying mechanisms despite their differences in cell type and lineage.

The aging-associated SBS5 signature accumulated in cerebellar GNs at rates comparable to the rate of SBS5 accumulation in cortical neurons and OLs (Fig. 2D). SBS5 is a “clock-like” mutational signature that accumulates in all cancers, cells, and tissues studied to date, and is the major driver of age-related mutation accumulation in cerebral cortical neurons (11), suggesting that it accumulates independently of cell division. SBS16, a transcription-associated mutational signature that also increases in cortical neurons with age (11), accumulated at a slower rate in GNs (p = 3.5 × 10^−4^, Tukey-corrected t-test; fig. S4). The higher burden of C to A mutations in GNs compared to cortical neurons or OLs is likely due to SBS8 and SBS38 signature mechanisms (Fig. 2, C and D). The etiology or underlying mechanisms of these two signatures remain to be fully elucidated, though SBS8 has been linked to deficient nucleotide excision repair of oxidative lesions (37, 38). SBS38 has been linked to ultraviolet light-associated melanoma (34), but the mechanism of its accumulation in GNs is unclear.

## sIndel signatures of cerebellar GNs differs from cerebral cortical neurons

**Fig. 3.**
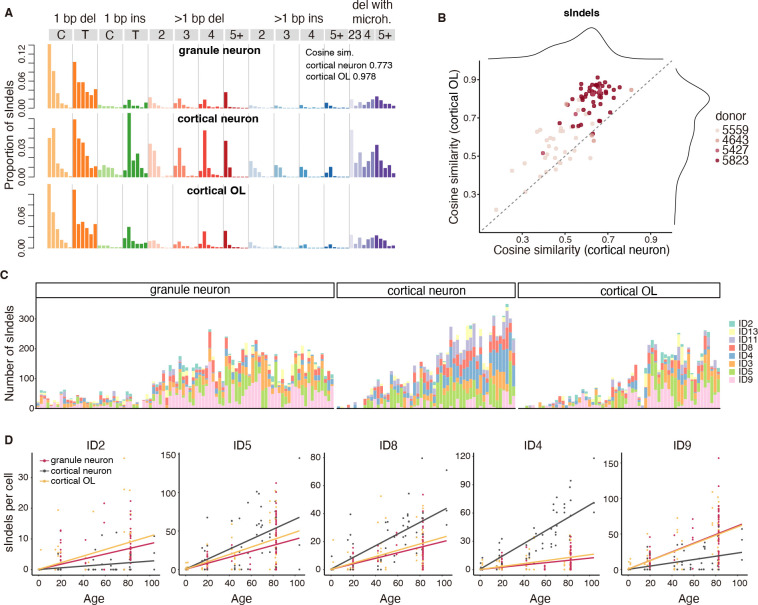
sIndel spectra of GNs show greater similarity to cortical OLs than to cortical neurons. A. sIndel mutational spectrum of the three cell types, with cosine similarities shown for GNs relative to cortical neurons and OLs . B. Cosine similarity of sIndel spectrum from each GN relative to cortical neurons and OLs. Points represent single GNs colored by subject. C. Decomposition of sIndel burden into COSMIC indel signatures in GNs, cortical neurons, and OLs. Subjects are ordered by increasing age from left to right. D. Contributions of the selected COSMIC indel signatures to the sIndel burden in GNs, cortical neurons, and OLs.

Analogous to our sSNV analysis, GNs accumulate sIndels with signatures more closely resembling OLs (cosine similarity 0.978) than cortical neurons (cosine similarity 0.773) (Fig. 3, A and B). GNs show a lower number of 1 base-pair insertions of thymine nucleotides compared to cortical neurons (p = 2.0 × 10^−9^, two-tailed Wilcoxon test; Fig. 3A). Further, ID4 accumulates at a much slower rate in aging GNs than in cortical neurons (p = 4.9 × 10^−10^, Tukey-corrected t-test; Fig. 3, C and D). ID4-like signatures have recently been identified as a hallmark of several neurodegenerative disorders (13, 39), and appear to reflect Topoisomerase 1 (TOP1)-mediated DNA damage (39, 40). ID2 and ID9 signatures on the other hand, accumulate at higher rates in GNs than cortical neurons (ID2: p = 4.8 × 10^−2^, ID9: p = 2.1 × 10^−2^), but at similar rates to OLs (ID2: p = 0.46, ID9: p = 0.99, Tukey-corrected t-test; Fig. 3, C and D). While the etiology of ID9 is unknown, ID2 is frequently observed in cancer tissues and is the result of DNA damage induced by replication slippage (34). In all, the sIndel data are consistent with our sSNV data supporting the idea that the mutational forces and mechanisms in cerebellar GNs are more similar to those in OLs than to cerebral cortical neurons.

## Genomic distribution and functional impact of GN somatic mutation

**Fig. 4.**
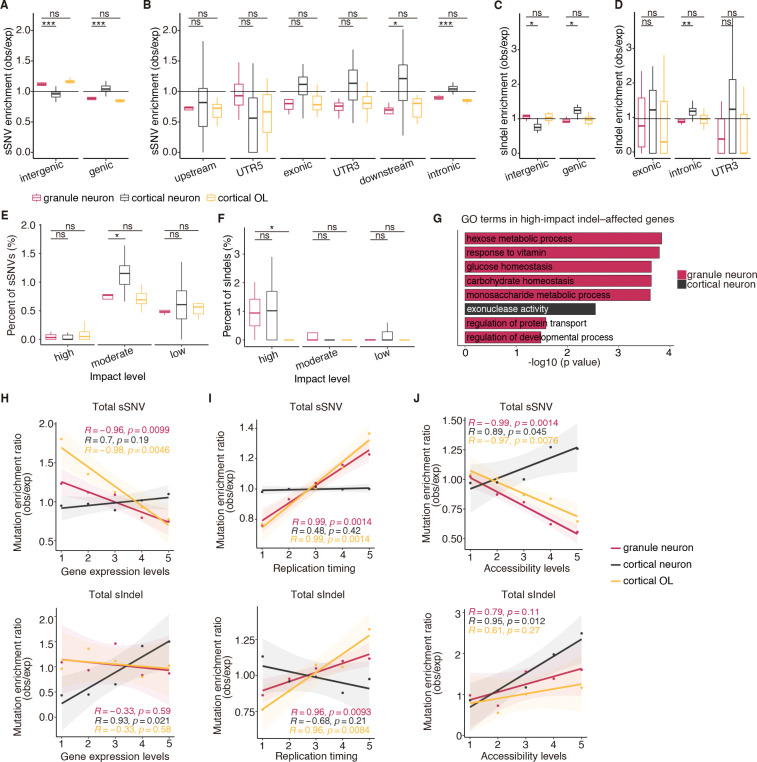
Enrichment analysis of sSNVs and sIndels in GNs. A–D. Genomic enrichment patterns of sSNVs (A, B) and sIndels (C, D) across cell types, displayed for intergenic and genic regions (A, C) and for detailed genic annotations (B, D). obs, observed; exp, expected (1,000 permutations). Significance denoted as: ns p ≥ 0.05, *p < 0.05, **p < 0.01, ***p < 0.001, ****p < 0.0001 using a two-tailed Wilcoxon test. E–F. Percentages of sSNVs (E) and sIndels (F) predicted as high-, moderate-, or low-impact in GNs, cortical neurons, and OLs. P value of two-tailed Wilcoxon test is provided. G. Gene ontology (GO) terms significantly enriched in genes affected by high-impact indels in granule and cortical neurons. H–J. sSNV and sIndel enrichment in relation to gene expression (H), replication timing (I), and chromatin accessibility (J). Gene expression and chromatin accessibility profiles for GNs were obtained from our current study and Ament et al.(28), respectively, whereas profiles for cortical neurons and OLs were taken from Jeffries et al. (49) and Ganz et al. (11). Replication timing was derived from ENCODE Repli-seq of 15 cell lines. Expected mutation density was computed from 1,000 permutations; points show the mean observed/expected ratio across subjects, and lines represent linear fits with 95% confidence intervals. Pearson’s correlation coefficient and P value are provided.

Patterns of mutational enrichment in GNs also more closely resembled mutational patterns in OLs than cortical neurons (Fig. 4, A to D). Cortical neurons show enrichment of sSNVs and sIndels in genic regions, whereas oligodendrocyte mutations are enriched in intergenic regions (11). GNs show enrichment of sSNV and sIndels in intergenic regions similar to OLs but distinct from cortical neurons (Fig. 4, A to D). Specifically, sSNVs were enriched in intergenic regions but depleted in exonic and intronic genic regions (Fig. 4, A and B). Similar patterns of intergenic enrichment were observed with GN sIndels (Fig. 4, C and D). Consistent with this pattern, GNs and OLs had fewer moderate-impact sSNVs than cortical neurons (Fig. 4E). Intriguingly, unlike OLs, GNs harbored a significantly higher fraction of high-impact sIndels largely localized in exonic regions (Fig. 4F and fig. S5A). The genes predicted to be affected are involved in metabolism-related pathways, including carbohydrate metabolism as well as neurotransmitter activity (Fig. 4G). Future studies should clarify the functional effect of these predictions.

The density of sSNVs is positively associated with transcription levels in cortical neurons but negatively associated with transcriptional levels in OLs (8, 11). Integrating our snRNA-seq data revealed negative correlations between GN sSNVs and gene expression, mirroring the pattern in OLs (Fig. 4H). sIndels followed a similar negative trend although this was not statistically significant (Fig. 4H). Leveraging replication timing profiles from 15 ENCODE Repli-seq cell lines, we found that similar to OLs, GNs showed an enrichment of sSNVs and sIndels in late-replicating genomic regions, an area of the genome thought to be transcriptionally silent (Fig. 4I). In line with their depletion in highly expressed genes, our GN sSNVs were also depleted in more accessible chromatin regions in a published dataset on human cerebellum (28) (Fig. 4J). This pattern matches that of OLs but again differs from cortical neurons (Fig. 4J). sIndels did not show a significant correlation with chromatin accessibility (Fig. 4J). Additionally, by dissecting mutational signature-specific enrichment patterns, we found SBS1, SBS8 and SBS38 contributing to sSNV enrichment in low-expression genes, late-replicating regions, and inaccessible genomic regions (fig. S5). Taken together, our data suggest that somatic mutations in GNs are enriched in inactive and less-highly transcribed regions of the genome, and that certain sIndels could have damaging effects affecting cellular metabolism.

## Lineage analysis of sorted single cerebellar GNs

Cerebellar GNs continue to be produced from precursor cells for some time after birth in animal models (17, 18), and the persistence of the EGL in the immediate postnatal period in humans (16) suggests postnatal GN generation as well, prompting us to examine cell lineage and the timing of GN production directly in human brain. We focused on our oldest subject (age 82.7 years) where we had generated 47 GNs sequenced at an average of 30X. To expand the number of cells and strengthen our analysis, we isolated and whole-genome sequenced an additional 131 GNs at an average of 10X coverage, a depth sufficient for accurate sSNV calling, supported by identical mutational spectra between 30X and 10X cells from the same individual (cosine similarity 0.992) (fig. S6, A and B). Further, as our SCAN2-based mutation calling may fail to detect some early clonal mutations present in the bulk control samples, we additionally generated 250X bulk WGS on matched cerebellum samples and called clonal mutations using MosaicForecast (41) to allow capture of additional clonal mutations (fig. S6B). Shared sSNVs identified clonally related neurons, while the count of unshared somatic sSNVs, since they accumulate in a highly linear, clock-like fashion with respect to postnatal age, could be used to approximate the age of the donor at the time of the most recent common ancestor (MRCA) for each neuronal lineage (11) by dividing genome-wide private sSNVs by the annual accumulation rate from GN aging trend line, and subtracting from the donor age at death following model-based offset correction (see Methods).

**Fig. 5.**
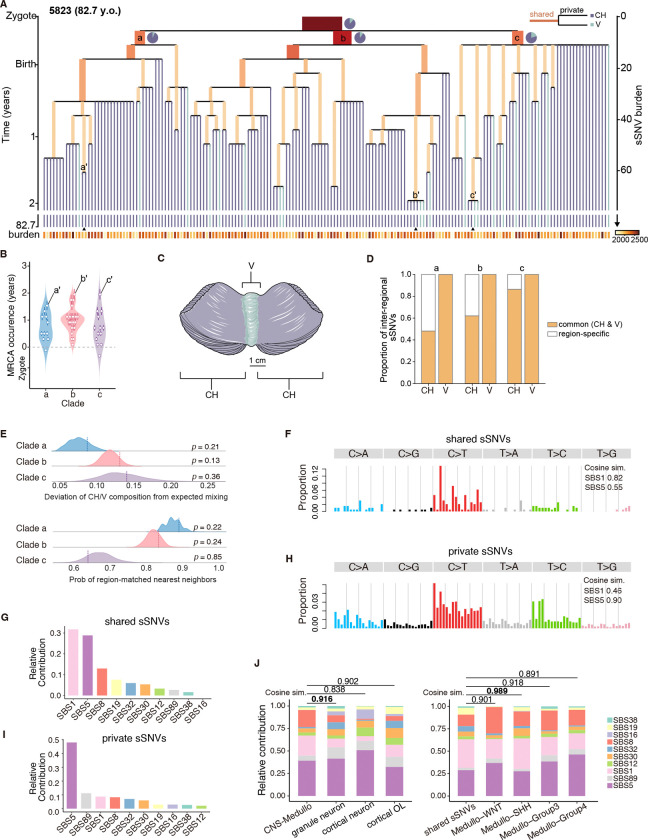
Single cell lineage tracing of cerebellar GNs. A. Early clonal phylogenies reconstructed for subject 5823 (82.7 year old; 178 cells). Tree depth reflects the number of accumulated sSNVs. Orange branches represent shared sSNVs; purple and light-blue branches depict private sSNVs unique to CH or V, respectively. Branch lengths are proportional to the number of sSNVs; branch width corresponds to the fraction of cells carrying the sSNVs. Pie charts categorize clones based on two regions (CH and V) at internal bifurcation. The annotation (bottom) shows the per-cell sSNV burden. Triangles mark the latest-born sub-clades (a’, b’, c’) derived from each of the three major clades (a–c). The developmental timing (left) was inferred from the linear mixed-effects model. B. Violin plots showing the distribution of the estimated MRCA occurrence time (in years from zygote) for clade a–c. Each point represents an inferred ancestral clone from the shared-mutation branches, with vertical error bars indicating the 95% confidence intervals derived from the linear mixed-effects model. C. Schematic depicting CH and V of adult human cerebellum. Adapted from Servier Medical Art (https://smart.servier.com) licensed under CC BY 4.0. D. Bar plots showing the proportion of common (orange) versus region-specific (white) sSNVs between CH and V cells within clade a–c. E. Measures of anatomical intermixing in the phylogenetic tree. Top: A region-mixing deviation index was computed to quantify how strongly the CH:V composition of inferred subclusters deviates from the overall CH:V ratio of each clade. Ridge density curves show the null distributions generated from 1,000 permutations of region labels, with vertical dashed lines indicating the observed values. None of the clades exhibited significant deviation from random mixing (permutation test: *p* = 0.21 for clade a; *p* = 0.13 for clade b; *p* = 0.36 for clade c). Bottom: Distribution of the probability that a cell’s nearest neighbors originate from the same anatomical region. Ridge curves show null distributions from 1,000 label permutations; vertical dashed lines indicate the observed values. No clade exhibited significant enrichment of region-matched neighbors beyond random expectation (permutation test: *p* = 0.22 for clade a; *p* = 0.24 for clade b; *p* = 0.85 for clade c). F–I. The mutational spectrum and contributions of COSMIC SBS signatures for shared (F, G) and private sSNVs (H, I). Shared sSNVs represent variants mapped to ancestral branches of the lineage tree, while private sSNVs represent terminal-branch variants unique to single cells. J. Signature contributions in medulloblastoma versus GNs, cortical neurons and OLs (left); and in shared sSNVs of GNs versus medulloblastoma subtypes (right). Cosine similarities measure pairwise contribution resemblance.

These clonal analyses reveal three major clades (a, b, c), each defined by distinct sets of clonal sSNVs, giving rise to the 178 GNs after fertilization (Fig. 5A and fig. S6C). We found that most ancestral clones across all three clades arose postnatally, with 45 out of 47 MRCA events dated after birth (Fig. 5, A and B), consistent with extensive GN formation postnatally. Notably, within clades b and c, the latest MRCAs for sub-clades b’ and c’ emerged as late as approximately two years of age (1.95 years, 95% CI: 1.76–2.15; Fig. 5, A and B). Moreover, we find that there is extensive spatial intermingling of clones that ultimately define the cerebellar hemisphere (CH) and vermis (V) (Fig. 5C), with cells from both CH and V being dispersed through all three clades a, b, c, and persisting even within those late-born sub-clades (a’, b’, c’) (Fig. 5, A to E).

To evaluate whether any region-enriched clones existed, we compared the proportion of common versus region-specific sSNVs between CH and V GNs. Although CH neurons showed more region-specific sSNVs (Fig. 5D), this is likely attributable to their larger representation in the lineage tree, which increases the probability of detecting hemisphere-restricted events. To account for this imbalance, we performed two permutation-based tests to assess whether any clade exhibited non-random anatomical enrichment (see methods), and both analyses showed no significant anatomical clustering across the three major clades (Fig. 5E).

Similar to our elderly 82.7 year old donor, we constructed a lineage tree of GNs from a 19.8 year old, albeit with fewer (41) cells, and identified several related clones that diverged around the time of birth or a few weeks postnatally (fig. S6, D and E). While the late birth of GNs is consistent with known timelines of cerebellar and granule cell development in humans (16, 17, 20–23), our findings suggest that widespread tangential migration of GNs or their precursors occurring late after birth can result in clones that are not restricted to anatomically defined areas of the cerebellum.

## Somatic mutation patterns in GN precursors mirror those in medulloblastoma tumors

Next, we asked if these clonal sSNVs that emerged early in life exhibited mutational signatures distinct from private sSNVs that accumulated later in GNs. We observed a pronounced enrichment of C>T mutations in clonal SNVs (Fig. 5F), with about 32% of clonal sSNVs attributed to SBS1—the largest relative contribution among the detected mutational signatures (Fig. 5G). Additional contributions from SBS5, SBS8 and SBS19 accounted for approximately 29%, 13% and 8% of clonal sSNVs respectively (Fig. 5G). In contrast, private sSNVs were dominated by SBS5 (48%), consistent with its established role as an age-related, clock-like mutational signature, and showed a reduced contribution from SBS1 (8.8%) (Fig. 5, H and I). These findings suggest that GNs experience different mutagenic forces in early development compared to aging.

We compared those somatic mutations arising in GN precursor cells to mutations occurring in cancer cells of the cerebellum to enlighten the cell of origin of cerebellar cancers. Granule cell precursors (GCPs) are the cell of origin of the sonic-hedgehog activated subtype of medulloblastoma (42–44), so we asked if mutational signatures observed in our GNs are present in medulloblastoma subtypes and to what extent. Using cosine similarity analysis of mutational signatures, we first found that GNs showed higher similarity (0.916) to a published medulloblastoma dataset (45), than cortical neurons and OLs (Fig. 5J). Then, we leveraged our lineage tree analysis of clonal sSNVs and compared mutational signatures to the subtypes of medulloblastoma. Interestingly, we found that clonal mutational signatures from GNs had the highest cosine similarity of 0.989 with the sonic hedgehog subtype of medulloblastoma (Fig. 5J), consistent with a likely origin of these tumors from GCPs. These analyses highlight the benefit of studying somatic mutations in normal cells as they can reveal shared mechanisms occurring in cancer cells, inform cell of origin of tumors, and ultimately treatment strategies for cancer.

## Discussion

Our data show that the mutational forces active in GNs show overlapping, but also some remarkably different patterns, from excitatory cerebral cortical neurons, despite GNs also functioning as excitatory neurons. Surprisingly, mutational patterns in GNs more closely resemble mutational patterns in OL glia than excitatory cerebral cortical neurons. The higher accumulation of cell-division-associated mutational signatures (SBS1 and SBS19) in aging GNs compared to cortical neurons is quite surprising, given that both neuronal types are considered postmitotic. This raises the possibility of ongoing low-frequency cell division events in the neuronal lineage in the aging cerebellum although we only found direct evidence for ongoing neurogenesis for about 2 years after birth. The absence of adult-born GNs from our 178 single cell analyses despite the accumulation of SBS1 mutations could reflect not capturing enough cells, or that GN birth past the early postnatal period does not occur. The latter case would prompt a re-examination of cellular processes driving SBS1 and SBS19 mutational signatures.

The sIndel mutation spectrum of GNs was also more similar to OLs than cortical neurons. Differences in somatic mutation rates and spectra seen in GNs may be linked to decreased transcriptional levels and complexity in GN and OLs compared to cortical excitatory neurons. Moreover, GNs have smaller nuclei and cell bodies compared to cerebral cortex excitatory pyramidal neurons (16, 46), which may reflect differences in chromatin packing, organization, and possibly the amount of DNA exposed to mutation or repair. Indeed, our data suggest that somatic mutations in GNs are depleted in more accessible chromatin regions, similar to OLs, but in contrast to larger excitatory neurons where the reverse relationship holds. Consistent with this notion is a preprint report that Purkinje cells, one of the largest neuronal types, accumulate somatic indels at twice the rate of cerebral cortical neurons (47). These differences suggest unexpected potential relationships between neuronal type, nuclear size, and chromatin organization to somatic mutation accumulation. Given the broad differences between GNs and cerebral cortical neurons, diverse neuronal types in the human CNS may be expected to have surprisingly diverse patterns of age-related mutation, which could have relevance for understanding the cell type-specific vulnerability so often seen in age-related neurodegenerative conditions.

The observation that CH and V neurons can originate from a single common ancestor as late as 2 years postnatally was surprising, because it indicates that daughter cells born this late postnatally must travel to different anatomical regions that could be separated by millimeter or even centimeter long distances, corresponding to hundreds to thousands of cell body lengths . It remains to be determined if there are specific regions of the developing cerebellum that harbor these late-born neurons, and if such migration is driven by the same signals that guide early-born GNs such as semaphorin-6A, brain derived neurotrophic factor (BDNF) and Bergmann glia fibers (19, 48).

Our finding that clonal sSNV mutational signatures closely resemble those of the sonic hedgehog activated medulloblastoma subtype supports the suggestion of GCPs as the cell of origin of this subtype of medulloblastoma. Moreover, it highlights the fact that mutational patterns in normal cells can be used to identify cell lineages of interest in cancer initiation. Identifying the mutational signature differences between normal cerebellar cells and malignant cells may reveal biological pathways that can be targeted to treat cancers of the cerebellum including medulloblastoma. This principle more broadly can be applied beyond cancers of the brain to other cancer types.

## Supplementary Material

Supplement 1

Materials and Methods

Figs. S1 to S6

Tables S1 to S3

References (50-65)

## Data Availability

New sequencing data generated in this study will be deposited in a public repository, with controlled use conditions set by human privacy regulations. All scripts will be made available on GitHub. Previously published PTA data for neurotypical controls were downloaded from dbGaP (phs001485.v3.p1) and NIAGADs (NG00162).
