# Supplementary material for: Somatic mutation in human cerebellum illustrates neuron type-specific patterns of age-related mutation
Source: bioRxiv. 2026 Mar 2:2026.02.27.708647. Preprint. [Version 1] doi: 10.64898/2026.02.27.708647 (PMC13001421; doi:10.64898/2026.02.27.708647)
Supplement: Supplement 1 [file NIHPP2026.02.27.708647v1-supplement-1.pdf]

bioRxiv preprint doi: https://doi.org/10.64898/2026.02.27.708647; this version posted March 2, 2026. The copyright holder for this preprint (which was not certified by peer review) is the author/funder, who has granted bioRxiv a license to display the preprint in perpetuity. It is made available under aCC-BY 4.0 International license.

Supplementary Materials

Materials and Methods

Human tissue sources

Frozen post-mortem brains were obtained from the NIH Neurobiobank at the University of Maryland Brain and Tissue Bank, and research on these de-identified samples was performed at Boston Children's Hospital with approval from the Institutional Review Board (S07-02-0087). All the neurotypical control subjects used in this study had no known clinical history of neurological disease or were listed as "unaffected control" in the biobank. Most of the neurotypical subjects were also used in prior studies (*7*, *11*) including the 82.7 year old subject 5823, where no clinical neurological diagnosis was known, and a neuropathological diagnosis included cerebral amyloid angiopathy and Braak stage III Alzheimer's Disease Neuropathologic change.

Nuclear isolation and sorting from frozen post-mortem brain

Fresh-frozen post-mortem human brain tissue, previously stored at -80°C, was dissociated on dry ice into a 7 mL Dounce homogenizer on ice with 1 mL of chilled nuclear isolation media (250mM Sucrose, 25mM KCL, 5mM $MgCl_2$, 10mM Tris-HCl pH 8, 0.1% Triton X-100, 0.2U/ul RNase inhibitor, protease inhibitor, and 1mM DTT). 50mg of tissue was homogenized on ice and carefully layered on top of 14 mL of chilled sucrose cushion buffer (1.8M sucrose, 3mM $MgCl_2$, 10mM Tris-HCl pH 8, 0.2U/ul RNase inhibitor, protease inhibitor, and 1mM DTT) in a 15 mL Falcon tube. The samples were spun in the centrifuge for 1 hour at 30,000 xg and the supernatant was then removed and discarded, leaving the pellet in about 100 µL sucrose cushion buffer. The pellet was then resuspended in 400 µL of chilled blocking buffer (0.8% Bovine Serum Albumin in 1X PBS, 0.2U/ul RNase inhibitor, protease inhibitor, and 1mM DTT), and the suspension was set on ice for 10 mins. After 10 mins, the suspension was filtered through a 40 µm filter into chilled 1.5 mL Eppendorf Lo-Bind tubes, which were then centrifuged for 10 mins at 900 xg. The supernatant was then removed, leaving 75 µL of total volume remaining. Blocking buffer was added to reach a total volume of 500 µL. The following antibody and nuclei stain were then added: anti-NeuN (Sigma Aldrich catalog# MAB377X) at 1:500, DAPI (ThermoFisher Scientific catalog# 62248) at 3:500. The antibody-stained nuclei were then incubated on a shaker at 4°C for 30 mins protected from light. The tubes were then centrifuged for 5 mins at 400 xg. The supernatant was removed, leaving 50 µL of nuclei pellet solution remaining. Blocking buffer was then added to reach a total volume of 500 µL. The nuclei pellet was resuspended, and the suspension was centrifuged and washed again at least an additional time with blocking buffer. The pellet was then resuspended and filtered through 40 µm filters into fresh, chilled 1.5 mL Eppendorf Lo-Bind tubes for sorting. NeuN+ nuclei were then identified by flow cytometry using forward scatter and side scatter gates (Fig. S1A) to refine the target population. snRNA-seq as described below was performed to verify purity of the target NeuN+ populations.

10x Genomics snRNA-seq

Nuclei from fresh-frozen post-mortem human brain tissue were prepared and sorted as described above. To prepare single nuclei for RNA sequencing, up to 13,000 nuclei were sorted directly into 1.5 mL Lo-Bind tubes containing Master Mix supplemented with Nuclease-Free

bioRxiv preprint doi: https://doi.org/10.64898/2026.02.27.708647; this version posted March 2, 2026. The copyright holder for this preprint (which was not certified by peer review) is the author/funder, who has granted bioRxiv a license to display the preprint in perpetuity. It is made available under aCC-BY 4.0 International license.

Water, as described in the 10X Genomics Chromium Single Cell 3' Reagent Kits v3.1 User Guide (CG000315 Rev E). The GEM generation & barcoding, post GEM-RT cleanup & cDNA amplification, and 3' gene expression library construction were performed as per the manufacturer's instructions. Libraries were then analyzed using the Agilent 4200 TapeStation System and sent to Psomagen for sequencing on the Illumina Novaseq X platform (2 x 150bp reads)

snRNA-seq analysis

Read alignment and gene expression quantification were performed using CellRanger (v8.0.1) with the human reference genome GRCh38-2024-A. Ambient RNA contamination was removed using CellBender (v0.3.0) (*50*), an unsupervised probabilistic model applied to raw count matrices to infer and subtract background noise, producing denoised expression matrices for downstream analysis. Subsequent quality control was performed using Seurat (v4.3.0) (*51*). Cells with ≤300 or ≥30,000 UMIs, ≤200 or ≥7,000 detected genes, or ≥10% mitochondrial UMIs were excluded. Genes detected in fewer than three cells were removed. Doublets were detected and excluded using scDblFinder (v1.16.0) (*52*). Expression values were normalized and log-transformed using "Seurat NormalizeData", scaled using "ScaleData", and dimensionality reduction was performed by principal component analysis on the top 2,000 variable genes selected using the "vst" method. Batch effects were corrected with Harmony (v1.2.3) (*53*), and clustering was performed using the Louvain algorithm via functions "FindNeighbors" and "FindClusters". The final cell types were annotated based on published cell type-specific gene markers (*27*, *28*).

Single-cell whole genome amplification and sequencing

NeuN+ nuclei from frozen post-mortem brain tissue were isolated as described above. Single nuclei were then sorted into individual wells of a 96-well plate containing BioSkryb PTA Cell Buffer. PTA was performed largely according to the manufacturer's protocol (ResolveDNA Whole Genome Amplification kit P00001-07292022) but with the following modifications. Briefly, cell lysis (addition of MS Mix to cells) was performed by mixing the plate at 1400 rpm for either 20 mins at room temperature (RT) per manufacturer's protocol, or 30 mins at 4°C to improve yield. After addition of the SN1 and SDX, the samples were incubated at RT up to 20 mins. Amplification with the reaction mix was performed as described in the manufacturer's protocol. Post-amplification products were then cleaned using a 2X bead prep using AmpureXP beads (Beckman Coulter Life Sciences catalog #A63882) and the yield was quantified using the Qubit 1X dsDNA, high sensitivity assay kit (ThermoFisher catalog #Q33231). Using single cell whole genome amplified (by PTA) product, libraries were constructed using KAPA HyperPlus Kits (Roche catalog# KK8514). See manufacture's protocol or (Bioskryb SkrybAmp for Ultra-low DNA Inputs; High Performance DNA Amplification from Small Samples P/N 100068). PTA product planned for 30X sequencing were sent to Psomagen Inc. for library preparation. For samples planned for 10X sequencing, libraries were prepared similarly. Briefly, no fragmentation was performed. The End Repair and A-Tailing reaction mix of Ultrapure Water, Fragmentation Buffer, ER/AT Buffer, ER/AT Enzyme was added to 300-500ng of PTA products in a final volume of 35ul using nuclease-free water. The End Repair and A-Tailing was then performed by incubating the samples at 65°C for 30 mins. To each sample, KAPA UDI Adapters (Roche catalog# 08861919702) were then diluted to 5µM using KAPA Adapter Dilution Buffer (Roche catalog# 08278539001). These adapters were then added to each sample along with nuclease-free

bioRxiv preprint doi: https://doi.org/10.64898/2026.02.27.708647; this version posted March 2, 2026. The copyright holder for this preprint (which was not certified by peer review) is the author/funder, who has granted bioRxiv a license to display the preprint in perpetuity. It is made available under aCC-BY 4.0 International license.

water, Ligation Buffer, and DNA Ligase. This was incubated at 20°C for 15 mins to ligate the adapters. Post-ligation products were cleaned using a 0.8X AmpureXP bead prep and 10X KAPA Library Amplification Primer Mix and 2X KAPA HiFi HotStart Ready Mix were added directly to the bead slurry prior to amplification. Samples were amplified with 10 PCR cycles and cleaned up using two consecutive 0.55X→0.8X double sided bead preps using AmpureXP beads. Quality of completed libraries were assessed using the Agilent 4200 TapeStation System. Libraries were then sequenced by Psomagen Inc. on Illumina Novaseq6000 or NovaseqX platforms (2 x 150bp).

Single-cell multiplex PCR and analysis

Quality control assay on amplified genomes was performed using a multiplex polymerase chain reaction (PCR) of four random genomic loci as previously reported (*8*, *12*, *54*). Briefly, 1µL of amplified post-PTA product was added to 20 µL of a PCR master mix containing 0.05 U/µL Phusion Hot Start II DNA Polymerase, 1µM Multiplex Primer Mix, 500uM dNTPs, and 1.25X Phusion HF Buffer. The reaction mixture was then placed in a thermocycler programmed to the following conditions: 94°C for 15 mins, 13 cycles of [94°C for 1 minute, 68°C for 1 minute with -1°C/cycle, 72°C for 1 minute], 35 cycles of [92°C for 1 minute, 55°C for 1 minute, 72°C for 1 minute], 72°C for 10 minutes and a 4°C hold. The multiplex PCR products were analyzed using a QIAxcel Advanced System as per the manufacturer's instructions and samples that showed 4 discrete bands <1000 bp were used for further analysis.

Bulk genomic DNA extraction and sequencing:

For newly generated bulk genomic DNA sequencing data, DNA extraction was performed on frozen post mortem human brain tissue using the DNeasy Blood & Tissue Kit (Qiagen catalog# 69504) as per the manufacturer's instructions.. Genomic DNA was sent to Psomagen Inc. for library preparation and sequencing.

Alignments for bulk and PTA data

BWA (v0.7.15) (*55*) was used to align the bulk WGS and PTA scWGS reads to the human reference genome (GRCh37 with decoy) to generate BAM format files. Picard Tools MarkDuplicates (v2.8.0) was run with duplicates marked. Indel realignment and recalibration of base quality scores were performed using Genome Analysis Toolkit (GATK) (v3.5) (*56*).

Quality control of scWGS

Sequencing depth was calculated from aligned BAM files using samtools (v1.15.1) (*57*). The evenness of single-cell genome amplification was evaluated using two metrics: median absolute pairwise difference (MAPD) and coefficient of variation (CoV). MAPD quantifies local amplification variability by computing the median absolute difference between log2-transformed copy number ratios of every pair of neighboring bins constructed with equal numbers of uniquely mapped reads. CoV captures genome-wide amplification variability and was calculated as the ratio of the standard deviation to the mean of bin-wise copy number ratios. Higher values of either metric indicate increased amplification unevenness.

Somatic mutation calling from PTA data

bioRxiv preprint doi: https://doi.org/10.64898/2026.02.27.708647; this version posted March 2, 2026. The copyright holder for this preprint (which was not certified by peer review) is the author/funder, who has granted bioRxiv a license to display the preprint in perpetuity. It is made available under aCC-BY 4.0 International license.

Single Cell ANalysis 2 (SCAN2, v1.1) (*8*) was used to detect sSNVs and sIndels from single-cell PTA data with matched bulk data. A cross-sample panel was first constructed using the "scan2 makepanel" with the following parameters: human reference genome GRCh37 with decoy sequences (--ref), dbSNP v138 common variants (--dbsnp), 1000 Genomes Phase 3 SHAPEIT2 phasing reference panel (--shapeit-refpanel), and metadata mapping each single-cell ID to its corresponding individual (--makepanel-metadata). Somatic mutation detection was subsequently performed on a per-individual basis using "scan2 call_mutations" with the following parameters: each PTA single-cell BAM file (--sc-bam), the matched bulk BAM file (--bulk-bam), and the cross-sample panel (--cross-sample-panel). For each cell, we obtained SCAN2 variant allele fraction (VAF)-based sSNV and sIndel calls, estimates of mutation detection sensitivity, and autosomal genome-wide burden from the corresponding scan2_object.rda file. Next, mutation-signature-based rescue was performed using "scan2 rescue" with the parameters: --rescue-target-fdr=0.01, and single-cell scan2_object.rda. Rescued mutation calls were used in detection of shared mutations and phylogenetic tree construction.

Linear mixed-effects modeling of mutation burden

To quantify age-associated accumulation rates of somatic mutations, we used the linear mixed-effects regression models from the lme4 (v1.1.36) R package. Both genome-wide and signature-specific mutation burden were modeled as continuous response variables. P values for pairwise comparisons of slopes were computed with the emmeans R package (v2.0.0), which utilizes Tukey-adjusted t-tests. To test the age effect of mutation burden in cortical neurons, cortical OLs and GNs from neurotypical individuals, we fitted the model $y_{ijk} = (\beta_j + \mu_{ij}) \times \rho_i + \varepsilon_{ijk};\ \rho_i = \alpha_i + c$, where y_{ijk} is the mutation burden in cell k from cell type j of individual i, β_j is the fixed-effect of age in cell type j, μ_{ij} is the individual-specific random effect on the age slope for cell type j following a normal distribution with mean 0 and variance σ, ρ_i is the chronological age of individual i measured from fertilization, α_i is the age of individual i at birth, $c = 268/365$ (268 days, median ovulation-to-birth interval (*58*)), ε_{ijk} is the cell-level residual measurement error following a normal distribution with mean 0 and variance τ. To obtain QC-corrected mutation burden estimates, we extended the mixed-effects model by including an additional covariate, θ_{ijk}, representing the QC metric (MAPD, CoV, sequencing depth, and mutation detection sensitivity), thereby re-estimating the age effect while accounting for technical variability.

Mutational signature analysis

To mitigate overfitting during mutational signature decomposition, we applied forward stepwise regression to preselect COSMIC v3.2 signatures (78 SBS and 18 ID signatures), as described previously (*59*). Signature inclusion was iteratively evaluated based on improvements in non-negative least squares fits, quantified by reductions in the sum of squared error (SSE) computed using the lsqnonneg function from the pracma R package (v2.4.4). The stepwise procedure was terminated when the SSE improvement fell below 30,000 for SBS signatures or 2,000 for ID signatures. This approach resulted in the selection of 10 SBS and 8 ID signatures, which were then refitted using MutationalPatterns (v3.14.0). Signature-specific somatic mutation burden was obtained by multiplying the contribution by the corresponding genome-wide mutation burden.

Annotation of genomic location and functional categories

bioRxiv preprint doi: https://doi.org/10.64898/2026.02.27.708647; this version posted March 2, 2026. The copyright holder for this preprint (which was not certified by peer review) is the author/funder, who has granted bioRxiv a license to display the preprint in perpetuity. It is made available under aCC-BY 4.0 International license.

Genomic annotations for sSNVs and sIndels were assigned using ANNOVAR (version updated in 2025March2nd) (*60*) based on the Func.refGene field. Variants were first classified into intergenic or genic categories. Genic variants were further subdivided into upstream (within 1 kb upstream of the transcription start site), 5′ UTR, exonic (coding sequence excluding untranslated regions), 3′ UTR, downstream (within 1 kb downstream of the transcription end site), splice-site (within intronic 2 bp of a splicing junction), and intronic regions. Variant counts were aggregated by cell type. To ensure robust estimation and avoid instability from sparse categories, only annotation categories with at least 20 total sSNVs or sIndels in any of the three cell types were included in the enrichment analyses shown in Fig. 4A–D. sSNV and sIndel functional consequences were annotated using SnpEff (v5.0) (*61*). Functional impact levels (High, Moderate, Low, and Modifier) were assigned using the first entry in the ANN field of SnpEff-annotated VCF files.

Permutation of mutation calls

Permutation-based background mutation sets were generated to control for potential biases introduced by trinucleotide context and the distribution of phaseable regions. In each permutation, the mutation calls were randomly shuffled within phaseable regions of the genome on a per-cell basis, while maintaining chromosome assignment and trinucleotide context. We performed 1,000 permutations of the sSNV and sIndel list for each cell using "scan2 permtool" with the parameters: the mutation calls (--permtool-muts), each single-cell ID (--permtool-sample) and 1000 permutations (--permtool-n-permutations).

Mutation enrichment analysis

We assessed enrichment and depletion of sSNVs and sIndels across genomic regions defined by gene expression, replication timing, and chromatin accessibility. Gene expression profiles for GNs were derived from in-house snRNA-seq data, whereas profiles for cortical neurons and OLs were obtained from Jeffries et al. (*49*) Replication timing data were obtained from ENCODE Repli-seq across 15 cell lines (*62*). Chromatin accessibility data for GNs were derived from a published human cerebellar snATAC-seq study (*28*), with corresponding profiles for cortical neurons and OLs sourced from our prior work (*11*).

For gene expression enrichment analysis, genes were ranked according to their expression levels and partitioned into five equal-sized expression groups. sSNV and sIndel densities were calculated separately for each group. Expected mutation densities were estimated using permutation sets (see "permutation of mutation calls"). Observed and expected counts were aggregated at the individual level, and enrichment ratio was calculated as the ratio of observed to expected mutation counts for each expression group. This analysis was performed separately for each cell type.

Replication timing signals were imported from bigWig files and partitioned into five equal-sized groups based on replication timing scores, ranging from early- to late-replicating regions. sSNV and sIndel densities were calculated for each replication timing group. For each cell type, individual-level enrichment ratios were first computed separately for each Repli-seq cell line in the same way as above and subsequently summarized by taking the median across the 15 cell lines.

For chromatin accessibility enrichment analysis, accessibility tracks of GNs generated in hg38 were converted to hg19 using UCSC LiftOver to ensure coordinate consistency across datasets. Accessibility signals were aggregated into non-overlapping 1kb genomic bins with

bioRxiv preprint doi: https://doi.org/10.64898/2026.02.27.708647; this version posted March 2, 2026. The copyright holder for this preprint (which was not certified by peer review) is the author/funder, who has granted bioRxiv a license to display the preprint in perpetuity. It is made available under aCC-BY 4.0 International license.

UCSC bigWigAverageOverBed. Genomic bins were classified into five accessibility groups, with bins showing zero signal designated as group 1 and bins with detectable accessibility divided into four equal-sized groups (2-5). Mutation enrichment ratios were calculated as described above.

Mutational signature enrichment analysis

Signature-specific enrichment analysis was conducted using the 10 COSMIC SBS signatures identified above (see "mutational signature analysis"). sSNVs overlapping each genomic group (e.g., gene expression, replication timing or chromatin accessibility group) were refitted to the selected signatures using MutationalPatterns fit_to_signatures function. Permuted mutation sets were processed identically to obtain expected signature contributions. Signature-specific enrichment ratios were calculated as the ratio of observed to expected mutation counts for each signature within each genomic group.

Gene Ontology analysis

Gene Ontology enrichment analysis was performed on genes with high-impact indels using GOseq (v1.54.0) (*63*) after controlling for gene length bias. GO terms with $P < 0.05$ are reported.

Detection of clonal sSNVs

For 30X PTA data, we used rescued sSNV calls generated by SCAN2. To increase sensitivity for detecting low-frequency somatic variants, we applied more lenient germline filtering criteria in matched bulk WGS data, including ≤1 low-quality alternative read (max.balt.lowmq), ≤ 10 total alternative reads (max.bulk.alt), and VAF ≤ 0.3 (max.bulk.af).

For 10X PTA data, a subset of variants was found to recur across multiple cells within the same batch, indicative of batch-related technical artifacts. To mitigate these artifacts, VAF-based sSNV calls were first aggregated across all cells within 10X PTA batch. For every candidate variant, mutation status was re-inferred from the raw BAM file of each cell using the Bayesian genotyper MosaicHunter (*64*) to estimate posterior probabilities of mosaic genotypes. Variants were subsequently projected into a matrix between recurrence ratio and 96-trinucleotide context, where recurrence ratio was defined as the proportion of cells within a batch harboring the variant. Variants exceeding a recurrence ratio threshold of 60%, informed by the recurrence ratio-context distribution, were classified as technical artifacts and excluded from rescued sSNV calls with lenient germline filtering criteria as described above.

For 250X bulk WGS data, sSNVs were called using MosaicForecast (*41*). Variants with mosaic_P ≥ 0.6 were retained as high-confidence calls, and variants overlapping gnomAD (*65*) were removed to exclude potential germline variants.

Finally, sSNVs identified from 30X PTA, 10X PTA, and bulk WGS data were integrated on a per-individual basis. Clonal sSNVs were defined as variants detected in at least two single cells, or in at least one single cell and a bulk sample. These clonal variants were re-evaluated using MosaicHunter in the same way as above to confirm the mutation status. To further reduce false positives introduced by lenient germline filtering, variants were filtered out if they showed excessive recurrence (recurrence ratio > 0.6) or insufficient allelic support (median VAF ≤ 0.4 across mutant cells). High-confidence clonal sSNVs were then assembled into a variant–sample (cell and bulk) genotype matrix per individual shown in Fig. S6B–C.

bioRxiv preprint doi: https://doi.org/10.64898/2026.02.27.708647; this version posted March 2, 2026. The copyright holder for this preprint (which was not certified by peer review) is the author/funder, who has granted bioRxiv a license to display the preprint in perpetuity. It is made available under aCC-BY 4.0 International license.

Phylogenetic tree construction

Phylogenetic trees were constructed for each individual through four steps. (1) Generation of the variant-cell genotype matrix. The variant-cell genotype matrix was extracted from the results described in "Detection of clonal sSNVs". The shared sSNVs among cells were kept for phylogenetic analysis. (2) Inference of clonal structure. To identify groups of sSNVs that co-occur across cells (hereafter referred to as mutation modules), the genotype matrix was subjected to hierarchical clustering using the pheatmap R package with complete linkage and binary distance. This step was used to reveal structured co-occurrence and mutual exclusivity patterns among sSNVs, providing an initial partition of sSNVs into nested candidate clone-defining sets. Variants incompatible with tree-like inheritance were subsequently pruned. Specifically, sSNVs were removed if they displayed random distributions across cells without forming stable co-occurrence patterns, or generated conflicts with nested inheritance, such that their presence-absence patterns could not be embedded into a hierarchical clonal structure. (3) Construction of phylogenetic tree. A lineage tree was then assembled by organizing the retained mutation modules into a hierarchical structure based on set inclusion relationships across cells. The tree was rooted at the earliest ancestral node, corresponding to the clone defined by the shared sSNVs present in the largest fraction of cells. Parent-child relationships between nodes were determined by the nested structure of mutation modules. Branch attributes were defined from the genotype matrix and the inferred hierarchy: branch lengths indicate the number of sSNVs acquired along that lineage; branch widths reflect the fraction of descendant cells carrying the corresponding sSNVs, providing clone prevalence within the cell population. (4) Developmental timing estimation and tree annotation. The shared sSNV count was extrapolated to a genome-wide burden after correcting for mutation detection sensitivity. These burdens were then used to infer the timing of ancestral events using the linear mixed-effects model (see "linear mixed-effects modeling of mutation burden"). Anatomical region labels were overlaid onto the phylogenetic tree for visualization.

The time to MRCA was calculated as the average inferred developmental time from zygote across cells. To assess whether cells from the CH and V exhibited non-random mixing in the lineage tree, we quantified the deviation of regional composition from that expected under random mixing. For each clade, we first calculated the global proportion of CH cells, and for each ancestral clone within the clade, we computed the absolute difference between the clone-specific CH proportion and the clade-level CH proportion. These differences were weighted by clone size and summed to generate a region-mixing deviation index for each clade. Statistical significance was assessed using 1,000 permutations in which regional labels were randomly shuffled among cells, and the observed deviation index was compared with the permutation-derived null distribution. We also estimated the probability that a cell's nearest neighbors arose from the same anatomical region. Nearest neighbors were defined as cells within the same ancestral clone. Region-matching probabilities were computed for each cell and averaged to obtain a clade-level metric. Statistical significance was assessed in the same way as above.

bioRxiv preprint doi: https://doi.org/10.64898/2026.02.27.708647; this version posted March 2, 2026. The copyright holder for this preprint (which was not certified by peer review) is the author/funder, who has granted bioRxiv a license to display the preprint in perpetuity. It is made available under aCC-BY 4.0 International license.

Supplementary Figures

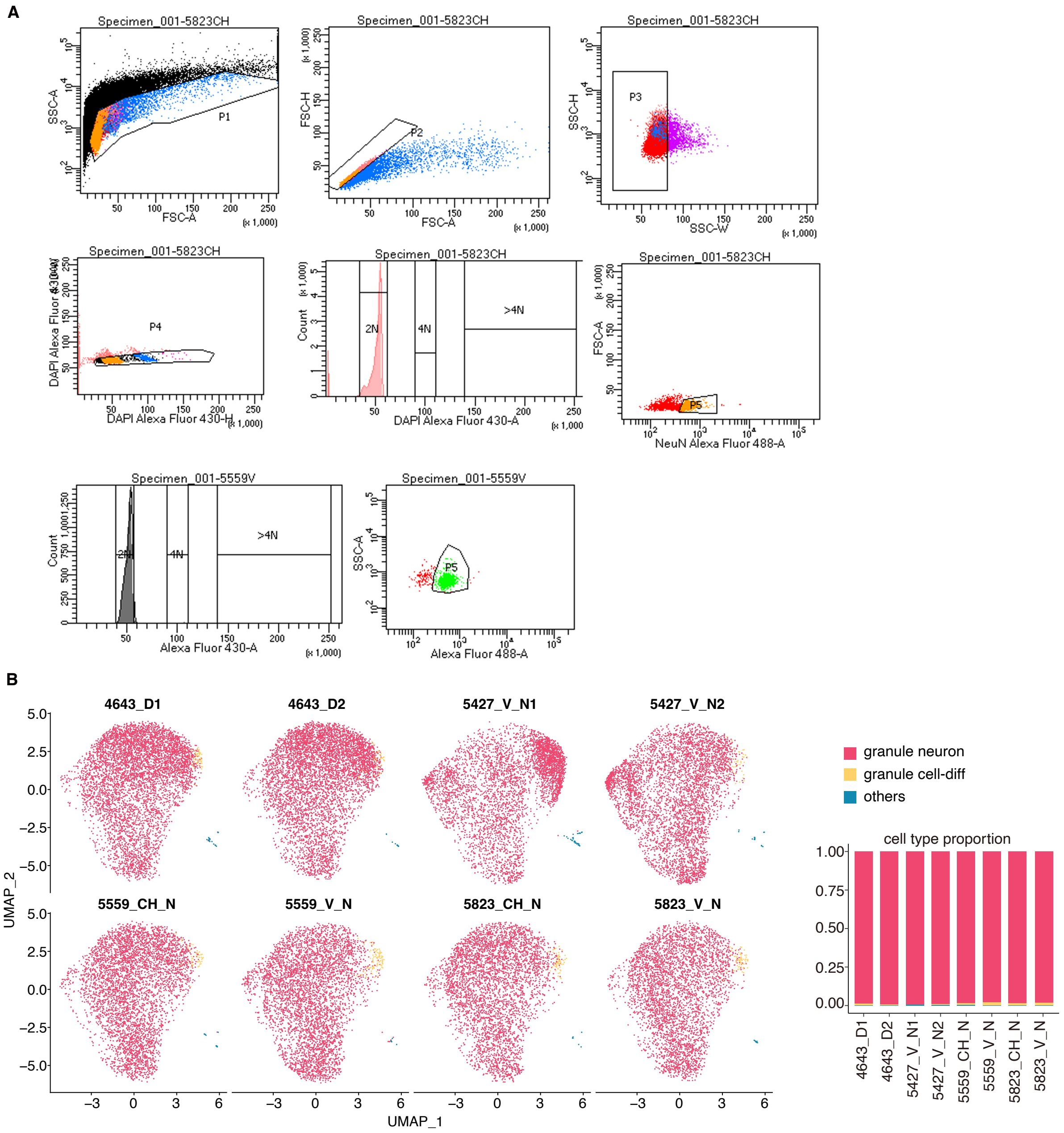

Fig. S1. FANS of neuronal nuclei from cerebellar brains and validation of purity.

A. FANS showing forward scatter and side scatter gates, DAPI+, 2N nuclei, and NeuN+ (P5) gates.

B. UMAP plots of snRNA-seq profiles (left) and cell-type composition (right) across samples.

bioRxiv preprint doi: https://doi.org/10.64898/2026.02.27.708647; this version posted March 2, 2026. The copyright holder for this preprint (which was not certified by peer review) is the author/funder, who has granted bioRxiv a license to display the preprint in perpetuity. It is made available under aCC-BY 4.0 International license.

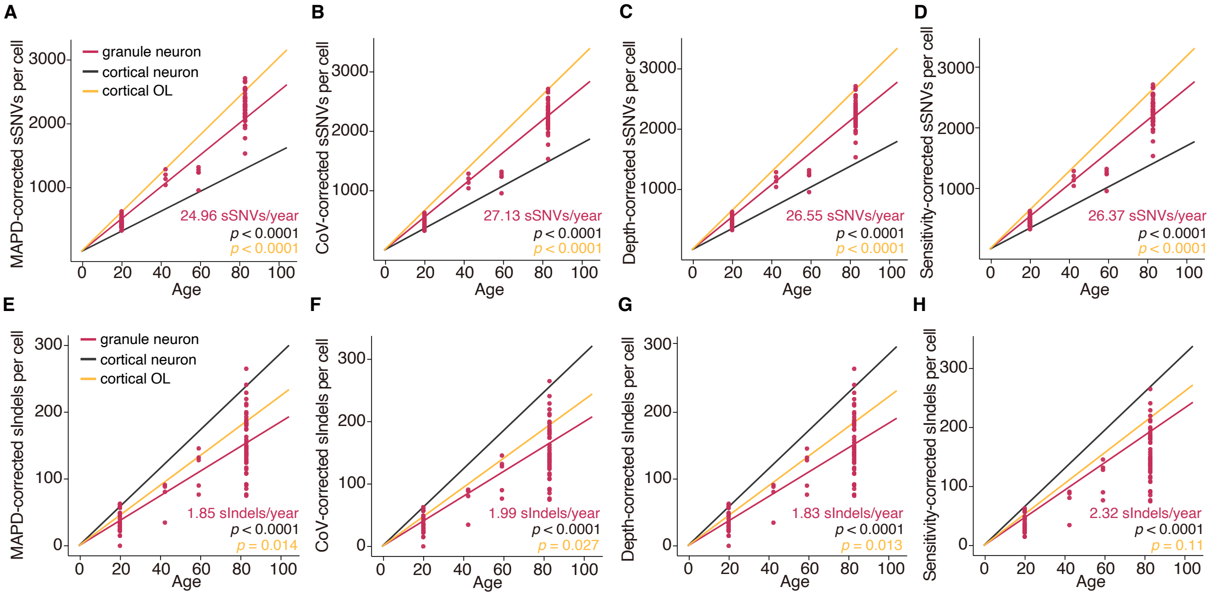

Fig. S2. sSNV and sIndel burden comparison after controlling for QC metrics.

A–H. sSNV (A–D) and sIndel (E–H) burdens in GNs, cortical neurons and OLs after correction for MAPD (A, E), CoV (B, F), sequencing depth (C, G), and SCAN2 mutation detection sensitivity (D, H). P values compare GNs with cortical neurons and OLs.

bioRxiv preprint doi: https://doi.org/10.64898/2026.02.27.708647; this version posted March 2, 2026. The copyright holder for this preprint (which was not certified by peer review) is the author/funder, who has granted bioRxiv a license to display the preprint in perpetuity. It is made available under aCC-BY 4.0 International license.

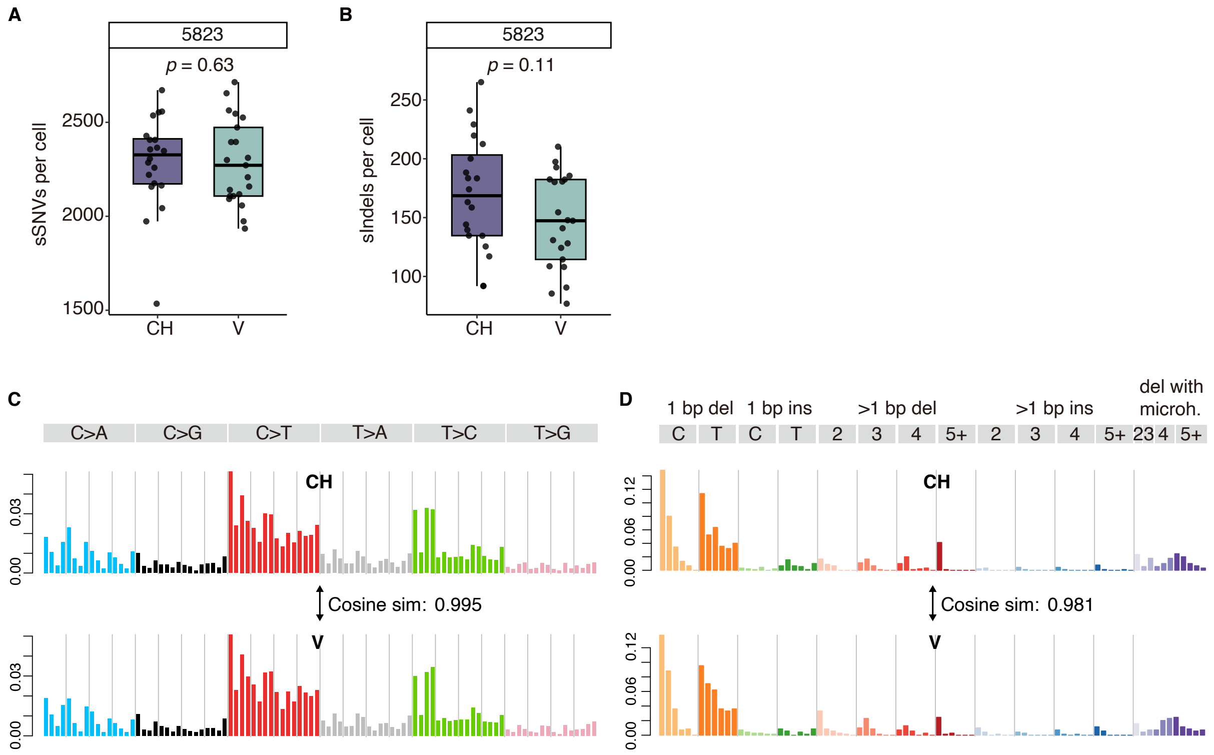

Fig. S3. GNs from CH and V show comparable somatic mutation profiles.
A–B. Comparison of sSNV (A) and sIndel (B) burden in GNs from the cerebellar hemisphere (CH) and vermis (V). P values are computed using two-tailed Wilcoxon tests.
C–D. Substitution (C) and indel (D) spectra of CH and V GNs. The text in the figure indicates the cosine similarities between regional profiles.

bioRxiv preprint doi: https://doi.org/10.64898/2026.02.27.708647; this version posted March 2, 2026. The copyright holder for this preprint (which was not certified by peer review) is the author/funder, who has granted bioRxiv a license to display the preprint in perpetuity. It is made available under aCC-BY 4.0 International license.

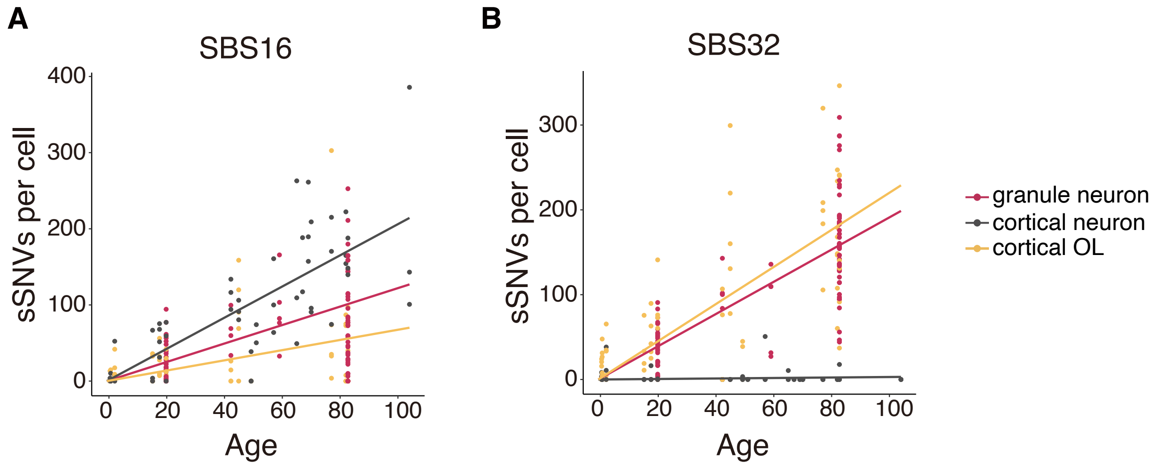

Fig. S4. Signature contributions across three cell types.
A–B. Signature contributions of SBS32 (A) and SBS16 (B) in GNs, cortical neurons, and OLs plotted against age.

bioRxiv preprint doi: https://doi.org/10.64898/2026.02.27.708647; this version posted March 2, 2026. The copyright holder for this preprint (which was not certified by peer review) is the author/funder, who has granted bioRxiv a license to display the preprint in perpetuity. It is made available under aCC-BY 4.0 International license.

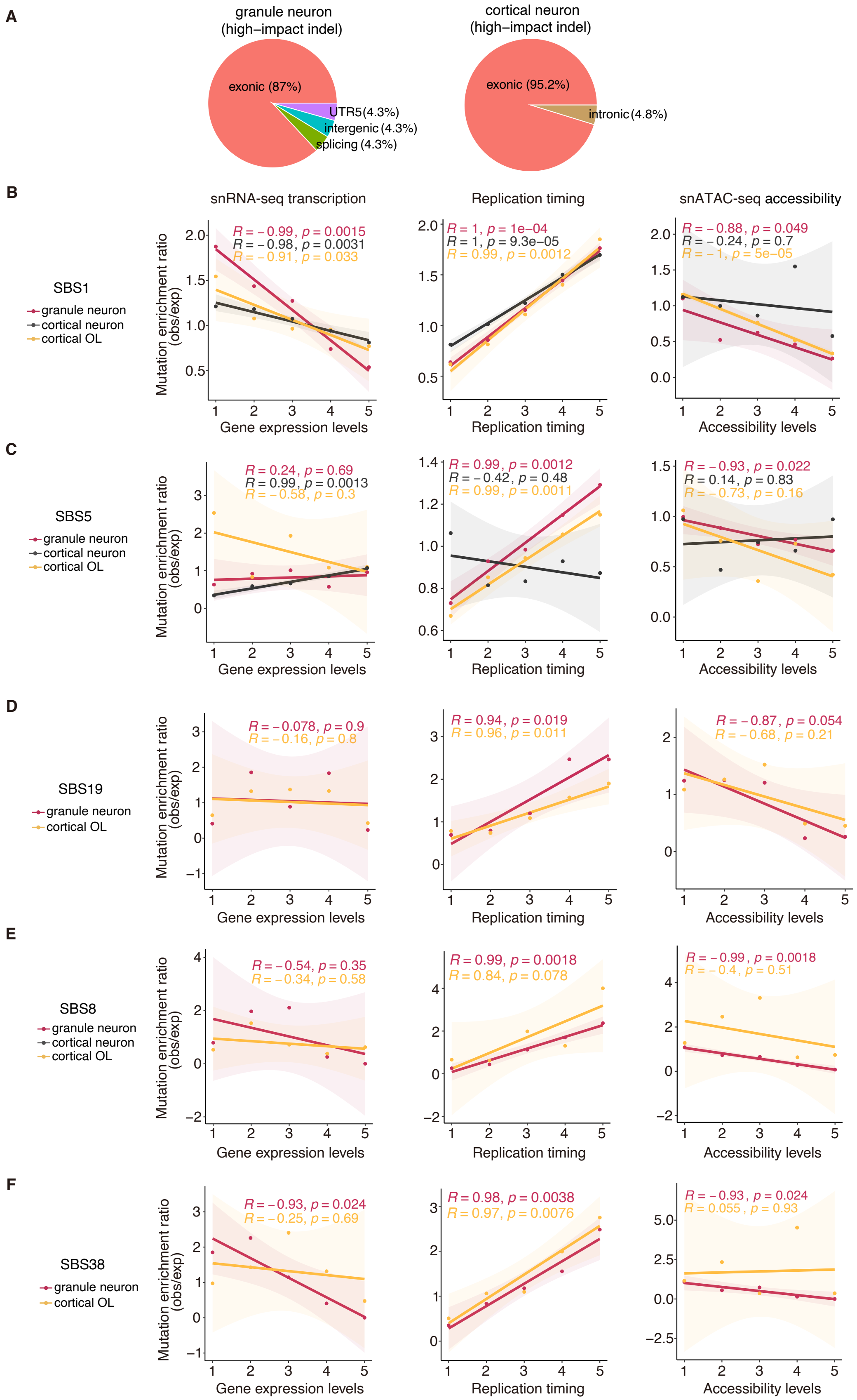

bioRxiv preprint doi: https://doi.org/10.64898/2026.02.27.708647; this version posted March 2, 2026. The copyright holder for this preprint (which was not certified by peer review) is the author/funder, who has granted bioRxiv a license to display the preprint in perpetuity. It is made available under aCC-BY 4.0 International license.

Fig. S5. Enrichment analysis of SBS signatures in GNs.
A. Pie charts showing the genomic distribution of high-impact indels identified in GNs (left) and cortical neurons (right).
B–F. sSNV enrichment for SBS1 (B), SBS5 (C), SBS19 (D), SBS8 (E) and SBS38 (F) in relation to gene expression, replication timing, and chromatin accessibility.

bioRxiv preprint doi: https://doi.org/10.64898/2026.02.27.708647; this version posted March 2, 2026. The copyright holder for this preprint (which was not certified by peer review) is the author/funder, who has granted bioRxiv a license to display the preprint in perpetuity. It is made available under aCC-BY 4.0 International license.

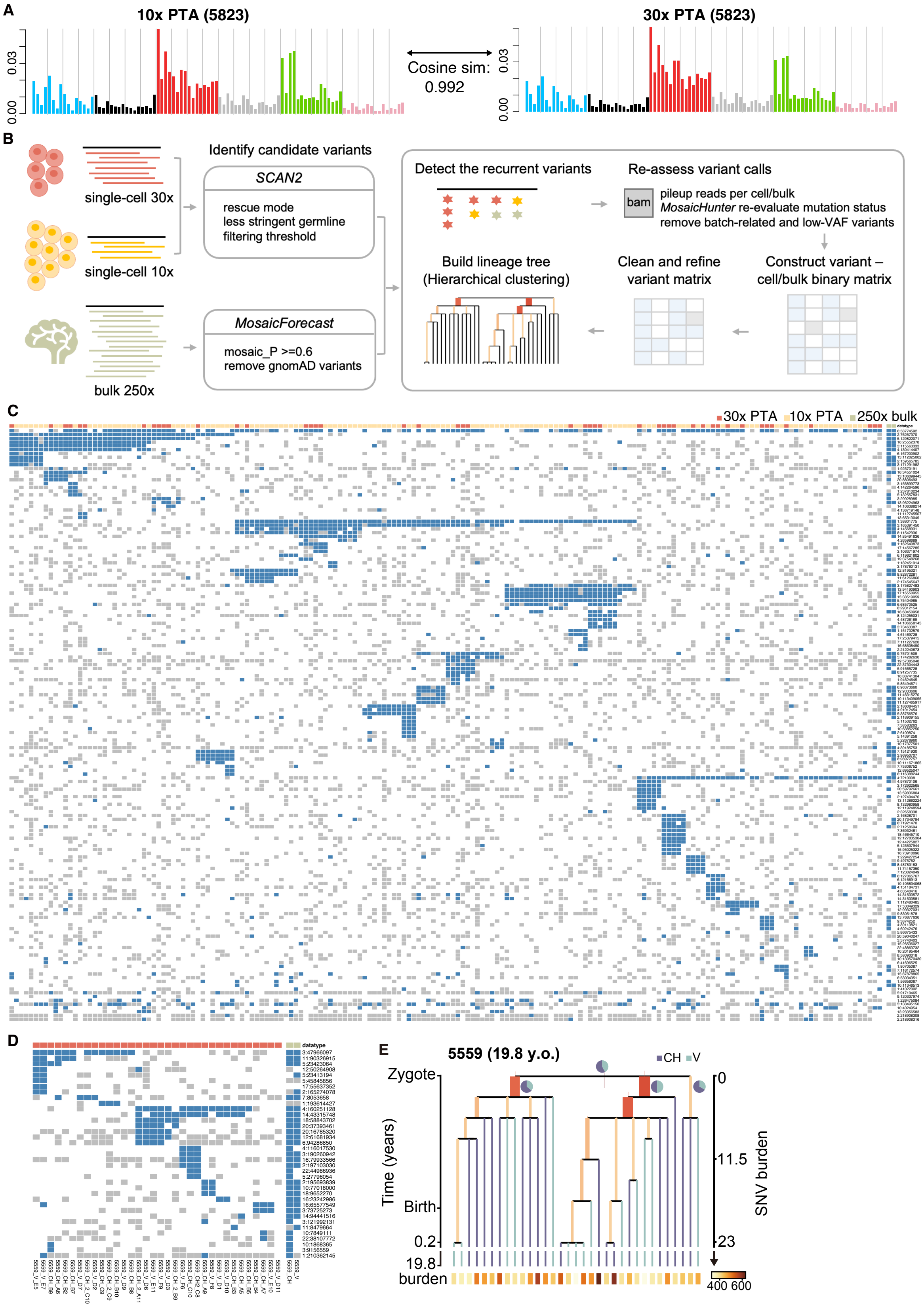

bioRxiv preprint doi: https://doi.org/10.64898/2026.02.27.708647; this version posted March 2, 2026. The copyright holder for this preprint (which was not certified by peer review) is the author/funder, who has granted bioRxiv a license to display the preprint in perpetuity. It is made available under aCC-BY 4.0 International license.

Fig. S6. Lineage tree analysis of GNs.
A. sSNV spectra of GNs from 30X and 10X PTA.
B. Schematic overview of the variant-calling and lineage reconstruction workflow. sSNVs from single-cell genomes (30X PTA and 10X PTA) were identified using SCAN2 with rescue mode and lenient filtering, while sSNVs from 250X bulk WGS were called using MosaicForecast. For each subject, variants from single-cell and bulk data were merged to detect recurrent mutations, which were subsequently re-evaluated per sample (single cell or bulk) using MosaicHunter to refine mutation status and remove batch-related and low-VAF artifacts. A variant-sample binary matrix was then constructed (blue: mutant; white: wild-type; grey: undetectable due to insufficient coverage) and cleaned to eliminate potential artifacts inconsistent with lineage structure. Hierarchical clustering was applied to reorder the matrix and generate the corresponding lineage tree.
C-D. Heatmaps showing the variant-sample binary matrices for subject 5823 (B) and 5559 (C), Each column represents a single cell or bulk (30X PTA: red; 10X PTA: orange; bulk 250X WGS: light green), and each row corresponds to a variant. Blue: mutant; white: wild-type; grey: undetectable due to insufficient coverage. Clonal blocks emerge as contiguous clusters of shared variants across cells, reflecting the underlying lineage structure.
E. Early clonal phylogenies reconstructed for subject 5559 (19.8 year old; 34 cells). CH, cerebellar hemisphere. V, vermis (vermis cells for this experiment on 5559 were performed immediately after CH cells in the same experiment, and hence may represent a mixed population of CH and V cells).
